# Different coding strategies for the perception of stable and changeable facial attributes

**DOI:** 10.1038/srep32239

**Published:** 2016-09-01

**Authors:** Jessica Taubert, David Alais, David Burr

**Affiliations:** 1 School of Psychology, University of Sydney, Australia; 2 Department of Neuroscience, University of Florence, Italy; 3 School of Psychology, University of Western, Australia

## Abstract

Perceptual systems face competing requirements: improving signal-to-noise ratios of noisy images, by integration; and maximising sensitivity to change, by differentiation. Both processes occur in human vision, under different circumstances: they have been termed *priming,* or *serial dependencies,* leading to positive sequential effects; and *adaptation* or *habituation*, which leads to negative sequential effects. We reasoned that for stable attributes, such as the identity and gender of faces, the system should integrate: while for changeable attributes like facial expression, it should also engage contrast mechanisms to maximise sensitivity to change. Subjects viewed a sequence of images varying simultaneously in gender and expression, and scored each as male or female, *and* happy or sad. We found strong and consistent positive serial dependencies for gender, and negative dependency for expression, showing that both processes can operate at the same time, on the same stimuli, depending on the attribute being judged. The results point to highly sophisticated mechanisms for optimizing use of past information, either by integration or differentiation, depending on the permanence of that attribute.

Detecting change, particularly in facial expression, is fundamental for perception. Adaptation to the prevailing level of any attribute – which leads to negative aftereffects – is thought to be a core mechanism for optimizing sensitivity to change^1^,^2^. Adaptation has been observed both for basic visual attributes, such as motion^3^ and orientation^4^, and also for more complex representations, such as numerosity^5^ and faces^6^,^7^,^8^.

On the other hand, in a constant but noisy environment, the system can profit by integrating successive views of images, which would produce positive rather than negative serial dependencies. Positive serial dependencies between successive images have been observed for some time. Repetition priming is a well known effect in perception of faces^9^, and many other attributes^10^,^11^,^12^,^13^. More recently, serial dependencies have been measured more directly, showing that the current image is often biased towards the previous, in orientation^14^, numerosity^15^ facial identity^16^ and, most recently, pulchritude^17^.

What determines whether assimilation or contrastive effects prevail, and how do the two opposing mechanisms interact? One factor is certainly stimulus conditions: strong, salient, high-contrast, long-duration stimuli tend to lead to negative aftereffects, while brief, less salient low-contrast stimuli lead to positive aftereffects^18^,^19^,^20^. But do they also depend on the type of information being encoded? For example, attributes that tend to be stable over time may be more prone to integration, while for changeable attributes, the system may gain more from contrastive adaptation to maximize sensitivity to change, especially if the change is functionally important. We tested this idea with perception of human faces, investigating, at the same time, perception of gender and expression. Gender is a stable attribute, which should not change with successive viewings, and should integrate. Expressions, on the other hand, are changeable, typically lasting between 0.5 and 4 seconds^21^, and important information is conveyed in the change. Although integration could be important for expression, it is also essential to differentiate successive images, to maximize the detection of change.

## Results

**Figure 1:**
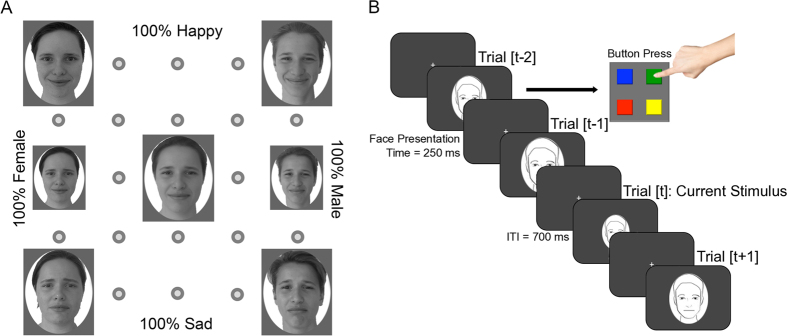
A representation of the 5 × 5 face-space produced from a single stimulus identity pair. The stimuli vary in expression (rows) and gender (columns). Note these stimuli are illustrative examples prepared for publication: the female/male stimulus pairs used in the experiment were taken from the NimStim imagebase.

With morphing techniques, we created a 5 × 5 two-dimensional gender/expression space from four different pairs of identities, one male and one female, each with a happy or sad expression. The stimuli varied smoothly from male to female along one dimension and happy to sad along the other (see example in Fig. 1). The stimuli were presented in pseudo-random order to nine subjects, who responded on each trial “male” or “female”, and also “happy” or “sad”.

**Figure 2:**
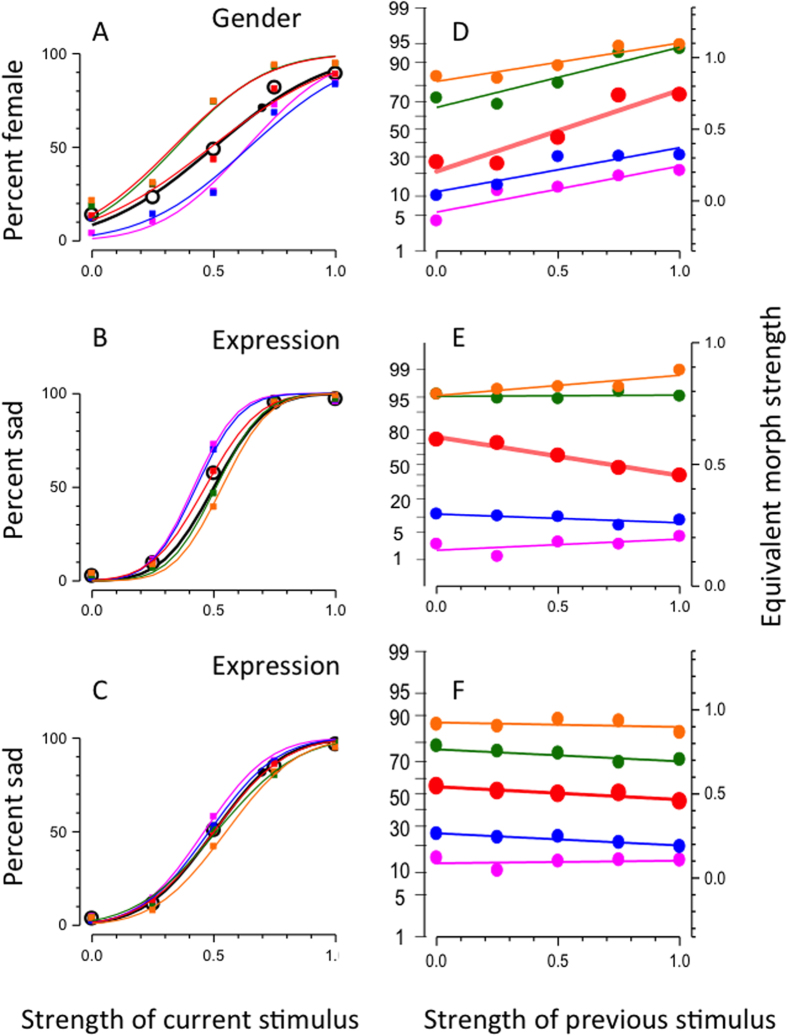
Serial dependencies in judging gender and expression. (**A**) Psychometric functions for judging gender as a function of morph strength. Open black circles with black psychometric function show average data, coloured squares data divided according to the morph-strength of the previous trial (orange 1; green 0.75; red 0.5; blue 0.25; magenta 0). The responses were fitted with cumulative Gaussian functions (colour-coded). The standard deviation of the fit to the average data was 0.32. (**B**) Psychometric functions for judging expression as a function of morph strength. The standard deviation of the fit to the average data was 0.15. Conventions as for **A**. (**C**) Psychometric functions for judging expression as a function of morph strength, using stimuli sampled at half-scale: the 0 and 1 strengths were the same as 0.25 and 0.75 in **B**. The standard deviation of the fit to the average data was 0.26. Conventions as for **A**. (**D**) Same data as **A**, plotted as percept response “female” as a function of morph-strength of previous trial, for five different morph-strengths of the current stimulus (from top to: orange 1; green 0.75; red 0.5; blue 0.25; magenta 0). Data are plotted on a probit scale, expressed as “percent female” on the left ordinate, and equivalent morph strength on the right (z-scores times the standard deviation the psychometric functions). The lines passing through the data show the best fitting regressions, yielding the following slopes: 0.32, 0.31, 0.57, 0.42 and 0.27. Calculations of weights were made from the curve measured at 0.5 morph-strength (red symbols and line), using eqn. 5. (**E**) Same data as **B**, plotted as percept response “sad” as a function of morph-strength of previous trial. All other details as for D. The slopes of the best-fitting regressions were, for current morph-strengths ranging from 1 to 0 were: 0.04, −0.03, −0.16, +0.005 and +0.05. (**F**) Same data as **C**, plotted as percept response “sad” as a function of morph-strength of previous trial. All other details as for D. The slopes of the best-fitting regressions were: −0.03, −0.07, −0.08, −0.07 and +0.01.

Figure 2A,B show the average results as a function of morph strength of the current trial, separately for gender and expression. Responses for both attributes varied smoothly with morph-strength, and were well fit with a cumulative Gaussian function (black curves). The other symbols and their fitted psychometric functions show results binned according to the morph-strength of the previous trial. It is apparent the curves are systematically displaced in both cases. However, the direction of the displacement was opposite for the two attributes: for gender they shift towards the previous stimulus (see legend); for expression they shift in the opposite direction.

The psychometric functions for expression (Fig. 2B) are steeper than those for gender, suggesting that, for this particular stimulus set, expression was easier to discriminate than gender. As this could influence the results, we repeated the experiment (on a partially new subject set), sampling expression at a finer scale (see methods), to yield psychometric functions of similar steepness as for gender (Fig. 2C). Again, the functions are separated with the same ordering as the functions in Fig. 2B, with full-strength expression.

Figure 2D–F show more clearly the effect of the previous stimulus on the response, plotting average response (on a Probit axis) as a function of strength of previous trial, separately for each morph-strength of the current stimulus. For gender (Fig. 2D), all curves show a strong positive dependency on the previous stimulus, resulting in positive slopes of the linear regressions. Although the strongest effects occur with the most ambiguous stimulus (the androgynous face: red symbols and lines), all regression lines have positive slope (slopes given below). As has been shown in the past for other visual attributes, such as orientation^4^ and numerosity^5^, the effects tend to saturate when the previous stimulus differs too much from the current stimulus. The responses for expression (Fig. 2E,F) also showed a strong dependency on the previous stimulus for the most ambiguous stimulus (morph strength 0.5). But here the dependency was clearly negative: if the previous face was happy, the current face was more likely to appear sad, and *vice versa*).

To calculate the magnitude and significance of the serial dependencies, we first converted the responses to equivalent morph strength, shown on the right-hand ordinate. This is a measure that takes account of the relationship between morph-strength and percent female or sad. Essentially, we used the black curves of Fig. 2A–C as a lookup table and read off the morph-strength corresponding to percent response (see methods for details). We estimated the magnitude of the effect of previous stimuli (relative to the effect of the current stimulus) from the slope of the best-fitting linear regression of these data. The slopes of the regressions for gender (respectively for current morph-strengths ranging from 1 to 0) were: 0.32, 0.31, 0.57, 0.42 and 0.27 (all significantly different from 0, two-tailed t-tests, p < 0.01). The regression slopes for expression, using the entire range of expressions were: 0.04, −0.03, −0.16, +0.005 and +0.05 (only the slope for morph-strength 0.5 was significant, p < 0.001); and for the reduced range of expressions, the slopes of the regressions were: −0.03, −0.07, −0.08, −0.07 and +0.01 (again, only the slope for morph-strength 0.5 was significant, p < 0.05). For further formal analyses, we considered only the responses to androgynous or neutral-expression stimuli, where the response to the current stimulus varies around 50%, free from floor or ceiling saturation effects.

**Figure 3:**
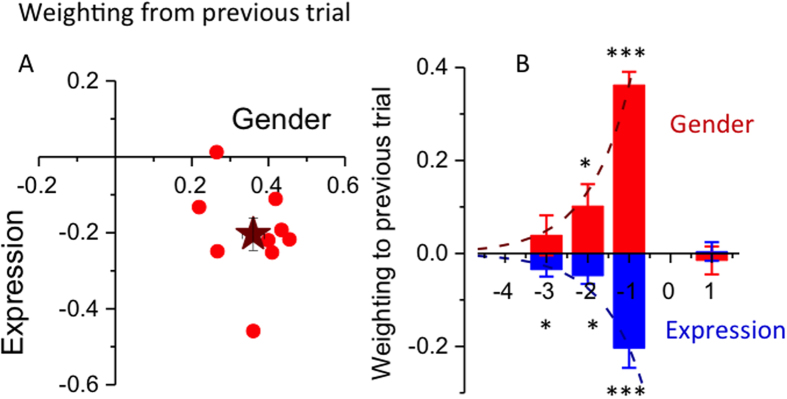
Weighting given to previous trials when judging gender and expression. (**A**) Weights of the previous trial for individual subjects, calculated from fitting the data at morph strength 0.5 with a linear regression (see red curves of Fig. 2D,E), and applying eqn. 5. Weights for gender are plotted on the abscissa, expression (full-strength) on the ordinate. The star shows weights calculated from data pooled over subjects with ±1 standard error bars. (**B**)Weights as a function of trial position, for gender (red) and expression (blue). The significance levels (one-tailed t-test) are indicated: *p < 0.05; **p < 0.001; ***p < 0.001. The values for *p* for gender were: < 0.0001, 0.041, 0.44, 0.57; expression: 0.0007, 0.021, 0.026, 0.54. The dashed curves are exponential fits anchored at the weight value for trial i-1 (equation 6).

We modelled serial dependencies with a simple Kalman-like filter^15^, where the response *R*_*i*_ to the current stimulus *S*_*i*_ (where *i* is trial number) is given by the weighted sum of the current stimulus, and the estimate of the previous stimulus 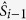.


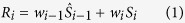



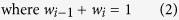



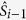
 is assumed to be approximated by *R*_*i*−1_, so the procedure is recursive. *w*_*i*_ can be considered the *Kalman weight.* The weight of the previous stimulus (*w*_*i−1*_) was calculated from the slopes of the best fitting regressions to individual subject data (equation 5 in methods). Figure 3A shows the data for 9 subjects, plotting weights for expression (full-strength) against those for gender. Dependency on the previous trial was strong and very consistent. For all subjects the weights were positive for gender, with an average value of 0.36. The weights for expression were negative for all but one subject, with an average value of −0.20. Both were highly significant (t tests, p < 10^−4^ for gender, p < 10^−3^ for expression). The weighting for half-strength expression was less than in the main condition (−0.10), but again highly significant (p = 0.001).

Figure 3B summarises the serial effects for gender and expression (full-strength) as a function of trial position. The effects of gender were significantly positive even for stimuli that preceded the test by two trials (p < 0.0001 for 1-back trials, p = 0.04 for 2-back trials); for expression, the effects remained significant even for three trials back (p = 0.0007, 0.021 & 0.026 for trials one-, two- and three-back). Importantly, there was no dependence on future trials for either attribute, showing that the correlations are causal, not artefactual. The dashed lines show exponential decay (expected because of the recursive nature of the model), anchored at the weights for trials preceding the current stimulus (equation 6). As the model predictions are based on the estimates of the previous trial strength, and those estimates are in turn influenced by those two trials ago, the process is iterative and should propagate over trials with an exponential decay. The data follow this prediction reasonably well.

## Discussion

This study demonstrates clear serial dependencies for gender and expression in the perception of faces, adding to previous reports of effects on face identity^16^ and attractiveness^17^. However, we show that not all attributes of the stimulus carry over from trial to trial in the same way: gender showed strong positive assimilation, while expression showed strong negative contrast effects. As the size of the stimuli varied over a 50% range during the experiment (±25% size change from trial to trial), it is unlikely that either the positive or negative dependencies were generated by local adaption to luminance, contrast or local, low-level features such as orientation.

Positive serial dependencies can be advantageous to vision, integrating previous with current estimates to improve signal-to noise ratios. In Bayesian terms, the previous stimulus acts like a *prior*, improving performance and minimizing “over-fitting” (the tendency of a system to follow variations due to noise, rather than real change^22^,^23^,^24^). In this sense it acts like a Kalman filter in systems control. Within this framework, perception can be considered a form of predictive coding^25^,^26^, where the previous estimate (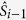 in equation 1) is the prediction to be combined with the current data.

However, the underlying assumption for all these theories is that the world does not change from trial to trial: while this is a reasonable assumption for gender, and for many other attributes such as identity, race and age, it is less reasonable for expression, which by its very nature is rapidly changeable: indeed, much of the information can be in the change. Assimilation with previous trials could often be detrimental, diluting sudden changes in expression that could convey important information. As change in expression can be fundamental for social interactions, the system may be optimized to detect change, using the basic mechanism to enhance change detection: adaptation, which causes to contrast effects. Adaption has been clearly demonstrated for many aspects of face perception under a variety of conditions^7^,^8^,^27^,^28^.

Distributed models of face perception propose separate representations of stable properties, such as identity and gender, from changeable properties such as emotional expression^29^,^30^. Both imaging and lesion studies suggest that stable features are processed in the fusiform gyrus, while changeable aspects are processed in STS^30^,^31^,^32^. Although not all agree with this dissociation^33^,^34^, if permanent and changeable aspects of face perception were subserved by separate neural circuitry, it is plausible that the different circuits would integrate information in a different way: stable traits profit from integration, whereas contrast-mechanisms optimize the detection of change.

The above discussion suggests that perception of gender and expression use *qualitatively* different integrating strategies. This is certainly plausible, given the evidence that they are analysed by different neural structures. Alternatively, however, assimilative integration may also occur with the perception of expression, but on a much shorter timescale, to allow for their changeable nature. There is a hint in our results that both assimilation and contrast effects may occur with expression, as the negative effects are much weaker and more variable than the positive effects. After converting to Kalman filter weights, the average weight for gender was 0.37 (*SD* = 0.08), compared with −0.19 (*SD* = 0.13) for expression (significant difference in magnitude, t(8) = 3.53, p < 0.01). There could be many reasons for these differences, but one possibility is that they may reflect the simultaneous actions of both assimilation and contrast effects with expression, in part cancelling each other out, and leading to greater variability between subjects. Expressions typically last between 0.5 and 4 seconds^21^. As each trial in the current experiment lasted 2 seconds on average, the duration was consistent both with constant and with changing expression in natural viewing. It would be interesting to repeat the study under conditions where the interval between pairs of trials could be shortened to sub-second levels, and also lengthened, to see if assimilation for expression may dominate at shorter time intervals.

Serial dependencies are usually considered to be automatic perceptual processes, rather than cognitive processes under voluntary control. They are often spatially specific^13^,^14^, automatic and do not lend themselves to intellectualizing^12^. However, the current study shows that although serial dependencies are not under conscious control, they can vary in how they optimize performance, within the same stimulus. For stable attributes such as gender the system fuses past with present information; for changeable attributes, such as expression, contrast effects are at work, presumably to facilitate detection of change.

An interesting question is how the perceptual system learns which aspects are permanent and which are transitory, and at what developmental stage this distinction is learnt. We have tested the theory only with faces, important naturally occurring stimuli that contain both permanent and changeable attributes, allowing us to test our prediction within the same single stimulus, at the same time. At this stage we cannot be certain that the principle will generalise to other stimulus types, or whether it is restricted to gender and expression. It would be interesting to repeat the experiment with other forms of stimuli, with permanent and changing attributes, to test how the principle generalizes to other non-facial stimuli. This should also allow us to study if and how integration strategies are learned, and whether they can change by manipulating the changeability of the stimuli.

## Methods

### Stimuli and procedure

We constructed four stimulus sets varying in both gender and expression from four different identity pairs (each pair comprised one male and one female, each with a happy and sad expression), chosen from the Nimstim imagebase^35^. All stimuli were gray-scale, with matched average luminance. For each male and female pair we first generated two cross-gender morph continua (separately for the happy and sad faces), extending from all-male through an androgynous stimulus to all-female, in 5 steps (female strength 0, 0.25, 0.5, 0.75, 1.0), defining the extremes of the expression morph-space. Using exactly the same procedure, for each level of gender, we morphed the 5 happy stimuli with the corresponding 5 sad stimuli (again in 5 steps) to produce 25 stimuli in total for each stimulus pair (Fig. 1, left panel). For the second experiment, expression was sampled at a finer scale (half that of the first study) by using the 0.25 and 0.75 morphs of the main experiment as extremes (relabelled 0 and 1), and interpolating between them and the 0.5 morphs to create the intermediate stimuli. The gender strengths remained the same as for the main experiment. All morphing was performed with *Psychomorph software*^36^, operating on 388 points positioned on specific facial landmarks.

For each of the identity pairs, the 25 stimuli were shuffled separately eight times and concatenated to produce a string of 200 images, to be displayed sequentially in a single session. Four sessions were run for each subject, randomizing order of the identity-pairs. Each trial began with a white fixation cross (10 pixels in width), drawn at the centre of a medium grey screen and visible for 700 ms before being replaced by a face stimulus. During the trial, a face was displayed for 250 ms under control of MATLAB on a CRT monitor (screen resolution 1024 × 768, 100 Hz refresh rate), viewed from distance of 57 cm. The base size of each stimulus was 12.75° in height, but size was randomized between trials, to minimize local adaptation effects: one third were 25% smaller, one third 25% larger.

Subjects were instructed to respond whether they perceived the face as male or female and also happy or sad, as quickly and accurately as possible, simultaneously on a 2 × 2 response box. We first piloted the idea of responding separately to gender or expression in different blocks but decided that both judgments should be made simultaneously in a single response on a four-button response box. Reaction times were similar for the two techniques (562 cf 573 ms for two pilot subjects), suggesting they had no difficulty in making the responses simultaneously. Consistent with this, the group average for all subjects with the four-button box was 590 ms (*SD* = 70 ms), giving an average time between trials of 1300 ms). On debriefing, subjects reported giving equal weight to both attributes, and not to have systematically decided on one before the other, a sensible strategy as both dimensions were randomly interleaved at various morph strengths so that there was never a reliably ‘easy’ dimension to judge first.

### Subjects

Nine subjects (5 female) participated in the main experiment, 8 of whom were naïve to the goals of the experiment (average age = 25.8 years, *SD* = 6). Two of these participated in the supplementary experiment, together with an additional 4 new subjects (average age = 29.2 years, *SD* = 4.5). All had normal or corrected-to-normal vision. All subjects gave written consent after being informed of the nature of the experiment (but not of its experimental aims). Experimental protocol was approved by the Human Ethics Committee at the University of Sydney, in accordance with the Code of Ethics of the World Medical Association (Declaration of Helsinki).

### Data analysis

We analysed individual data for each subject, and also pooled data, separately for gender and expression. We first plotted psychometric functions, the probability of reporting “female” or “sad” as a function of gender or expression strength. Data were fitted with cumulative Gaussian functions (Fig. 2A,B) of variable mean and standard deviations.

The data of Fig. 2D–F are plotted on a probit scale (indicated on the left ordinates), which is the inverse of the cumulative normal distribution. Data are expressed both as percent “female” or “sad”, and as “equivalent morph strength”: this is the morph-strength that corresponds to that percentage female or sad, derived from averaged data. Essentially, for any given percentage value of the black curves of Fig. 2A–C, read off the corresponding morph-strength from the abscissa. In other words, invert the cumulative gaussian psychometric function. In practice, this is achieved by multiplying the probit of the probability by the standard deviation of the fitted psychometric functions, and adding the mean.


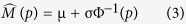


where 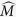 is the equivalent morph strength, μ and *σ* the mean and standard deviation of the best-fitting cumulative gaussian, and Φ^−1^ the probit function (inverse cumulative gaussian).

We modelled serial dependencies with the Kahlman-like filter of eqn. 1^15^. The slopes of the fitted regressions (ρ: red lines in Fig. 2D–F) estimate the value of *w*_*i−1*_ relative to *w*_*i*_:


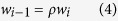


As the weights sum to unity (equation 2):


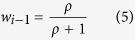


The data points of Fig. 3A were obtained from the slopes of the regressions of the individual subjects, using the standard deviations calculated from each subject’s data. The star in Fig. 3A, and all points in Fig. 3B were obtained from the pooled data. The standard errors, and the significance tests, were obtained from the individual subject data.

The exponential decays of predicted weights 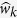 of Fig. 3B are negative exponentials passing through the value of *w*_*i−1*_:


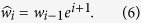


## Additional Information

**How to cite this article**: Taubert, J. *et al*. Different coding strategies for the perception of stable and changeable facial attributes. *Sci. Rep.* **6**, 32239; doi: 10.1038/srep32239 (2016).
